# Multifunction plasma genus holographic antenna for near/far-field focusing using a single structure

**DOI:** 10.1038/s41598-026-47001-y

**Published:** 2026-04-20

**Authors:** Nermeen A. Eltresy, Hend A. Malhat, Saber Zainud Deen, Mona M. Badawy

**Affiliations:** 1 Microstrip Department, Electronics Research Institute, Cairo, 12622 Egypt; 2 Faculty of Electronic Engineering, Menoufia University, Menoufia, Egypt

**Keywords:** Plasma, Beam steering, Multifunction antenna, Hologram Surfaces, Engineering, Physics

## Abstract

This paper introduces a plasma-based genus holographic reflectarray antenna (GHRA) for reconfigurable electromagnetic wave manipulation at 10 GHz. It introduces unprecedented dual-mode near-field (NF) focusing and far-field radiation within a compact 31.5 × 31.5 cm^2^ planar surface. By leveraging plasma frequency modulation, the GHRA dynamically tunes surface impedance from 267 Ω to 579 Ω, enabling precise electronic beam steering from θ =  − 40° to + 40° with a peak gain of 28.7 dBi at broadside and 17.4 dBi at extreme angles. A novel chessboard arrangement facilitates dual-beam radiation from a single activated surface, producing beams at (θ₁ = 10°, φ₁ = 0°) and (θ₂ =  − 30°, φ₂ = 0°) with gains of 21.2 dBi and 20.6 dBi, respectively. For both activated surfaces, dual beams with different directions are radiated. Beam1 at (θ_1_ = 10°, θ_2_ =  − 30°, φ_1_ = 0° with peak gain of 18.7 dBi and Beam2 at (θ_3_ = 10°, θ_4_ =  − 30°, φ_1_ = 0°) with peak gain of 18.7 dBi. The GHRA’s dual-surface design supports simultaneous NF focusing at R_o_ = 63.6 cm, yielding a tightly focused spot (3.25 cm × 3.75 cm) with a side-lobe level of -17.4 dB, and bidirectional far-field radiation with a 24.7 dBi gain. This innovative plasma-based technology, validated through simulations, offers unparalleled flexibility for applications in medical therapy, satellite communications, and phased-array radar, redefining the boundaries of antenna performance and adaptability.

## Introduction

Non-destructive testing (NDT) using microwave imaging is an emerging technology in advanced industrial and commercial applications^1^. It produces high-resolution images of complex structures, human tissues, concealed weapons, and subsurface exploration detection. Microwave imaging is employed in different applications such as security screening, remote sensing, healthcare, and industry^2^. High-gain, wideband antenna arrays are widely employed in microwave imaging due to their advantages, such as high resolution, penetration depth, and adaptability to different media^3^. A Genus antenna array (GAA) is a specific arrangement of elements designed to achieve desired radiation characteristics, including beam direction, shape, gain, and bandwidth. It is designed to improve signal reception or transmission through electromagnetic waves (EMWs), focused in a specific direction. GAAs are applied in applications where beam-steering, reduced interference, and efficient communication links are required^4,5^. Reconfigurable antennas can adjust their frequency, radiation pattern, or polarization, presenting flexibility in wireless communication^6^. There are different mechanisms for obtaining reconfigurable antennas, such as electrical, optical, and materials-based reconfigurability. Reconfigurable antennas are able to adjust their functionality based on mission requirements, low cost, easy integration, and support multiple wireless standards^7^. Plasma material is a versatile medium that possesses distinct properties as both a conductor and a dielectric; consequently, it can be used as a reconfigurable material^8^. The procedures employed for plasma generation vary depending on the required application. The most utilized methods include DC ionization, RF ionization, and laser excitation in gas plasma generation^9^. A plasma antenna consists of a dielectric material, such as a glass tube, which is filled with low-pressure noble gas, such as argon, neon, or xenon. The radiation characteristics of a plasma antenna can be electrically controlled through the applied ionizing voltage. These antennas can be rapidly energized and de-energized within a fraction of a second, thereby preventing any degradation of the transmitted signals^10^. A low-profile, broadband reconfigurable plasma antenna, designed for naval communications in the VHF and UHF bands (30–512 MHz), is presented in Ref.^9^. The antenna integrates a metallic inverted-dicone structure (operating from 100 to 512 MHz) with eight symmetrically arranged plasma tubes that, when ionized, function as conductive elements to extend coverage down to 30–100 MHz. It achieves a VSWR ≤ 2.5 across the entire band, along with stable radiation efficiency and reconfigurability through plasma excitation. A reconfigurable plasma-based reflectarray and transmitarray antenna, enabling electronic beam steering by controlling the argon plasma frequency through applied voltage, is presented in^10^. The 13 × 13 reflectarray operates at 12 GHz, achieving a peak gain of 24.3 dBi with beam scanning from 10° to 70°, whereas the 9 × 9 transmitarray operates at 15 GHz with peak gain and beam scanning from − 40° to + 40°. In^11^, a plasma-based circularly polarized (CP) magneto-electric (ME) dipole antenna is proposed as the elemental unit for time-modulated arrays (TMAs), achieving a wide impedance matching bandwidth of 2.75 GHz and a peak gain of 9.1 dBi. In Ref.^12^, efficient, reconfigurable linearly polarized (LP) to CP wave conversion and beam focusing using plasma ionization is demonstrated. An 8 × 8 intelligent metasurface generates left-hand CP from the upper surface and right-hand CP from the lower surface with a peak gain of 13.2 dBi and an axial ratio (AR) bandwidth of 2.1 GHz. A study of phase quantization effect on the array performance of 13 × 13 elements to reduce the ionization complexity of the IRS is introduced, with a gain reduction to 18.5 dBi for 3-quantization levels with 0.9 dB loss.

Hologram antennas (HA) are structures able to radiate a focused beam of EMWs with desired characteristics by applying a proper excitation wave^13^. It applies principles from optical holography to microwave and millimeter-wave frequencies. The HA consists of two main components: (1) the holographic surface, a metasurface made up of subwavelength elements such as patches, slots, or pins arranged on a dielectric substrate with spatially varying impedance. (2) The feed, which generates a reference wave to excite this surface^13^. When the reference wave propagates across the metasurface, it interacts with this hologram pattern (varying surface impedance), causing controlled leakage of energy that reconstructs the desired radiation pattern. This is achieved by applying a technique called surface impedance modulation on the metasurface, which involves changing the size of the patches on the dielectric-covered ground plane according to a predetermined phase distribution^14^. HAs offer the advantages of low profile, light weight, high gain, and narrow beam width^15^. In addition to that, they can perform beamforming and frequency scanning with different polarizations, avoiding the need for complex feeding networks^16^. HAs play a crucial role in various applications, such as near-field EMW signal transmissions, imaging, beam shaping, and radar cross-section reduction^17–21^. In^22^, a colorful image consisting of three major colors was created using a multi-color metasurface hologram, by modulating the wave front capacity of each antenna at different operating frequencies. One-dimensional (1D) and two-dimensional (2D) hologram antennas were employed to control the propagation of surface EMWs. The 1D hologram antennas have a reduced number of unit cells compared to the 2D hologram antennas, which consist of a larger number of unit-cell elements with varying sizes. The advantage of using 1D hologram antennas lies in the reduction of computational complexity in surface impedance calculation and the overall antenna size. A comparison between 1 and 2D hologram antennas was presented in Ref.^14^. Traditionally, most antennas are designed for applications in the far field. However, the Fresnel zone presents a unique opportunity to transmit power in a concentrated beam. In this region, the aperture behaves similarly to a lens, allowing for the concentration of light into a beam with a minimal spot size at the focal distance^23^. Using lens geometry, the appropriate phases for the array elements can be calculated^24^. The ability to focus microwave energy in the Fresnel zone has garnered significant interest in various fields such as medical imaging and therapy, remote sensing, and wireless power transfer^25^. Fresnel zone focusing plays a crucial role in microwave-induced hyperthermia, where EM fields are confined to cancerous regions while minimizing harm to surrounding tissue. In remote sensing, focusing apertures are essential for providing precise sensing information, such as monitoring the temperature of food products in a production facility, without the need for direct contact.

In this paper, a reconfigurable plasma GHRA for single and multiple beam operation at 10 GHz is presented. The design of a unit-cell element with controlled plasma ionization voltage is studied. The GHRA’s dual holographic surfaces control beam direction and focal distance for both far-field (FF) steering and near-field (NF) focusing, modulated by ionization voltage. A focused beam at a certain distance away from the aperture of the proposed array is obtained. The radiation characteristics of GHRA are investigated by phase correction of array elements without using complex microwave feeding networks. Full-wave CST Microwave Studio (MWS) simulator is used to numerically analyze the proposed GHRA for different beam directions^26^.

## Characteristics of plasma material

Plasma is the 4th state of matter and is produced by ionizing inert gases. Plasma species primarily consist of electrons, positive ions (e.g., Ar⁺), and neutral gas atoms; however, for microwave frequencies, the electromagnetic response is dominated by the electron density and collisions, with ions contributing negligibly due to their much higher mass. Assuming plasma as a cold, collisional, and unmagnetized medium with dispersive properties given by the Drude model^13^:

1 $${\varepsilon }_{r}\left(\omega \right)=1-\frac{{\omega }_{p}^{2}}{{\omega }^{2}+i{\nu }_{p}\omega },$$

where ${\omega and \varepsilon }_{o}$ are the angular frequency, the free space permittivity, and ${\nu }_{p}$ is the electron-neutral collision frequency. The plasma angular frequency is given by^13^:

2 $${\omega }_{p}=\sqrt{\frac{{n}_{e}{e}^{2}}{{\varepsilon }_{o}{m}_{e}}},$$

where *n*_*e*_*, e,* and *m*_*e*_ are the electron density, charge, and mass. The plasma conductivity is tuned via applied voltage, which controls the electron density of the ionized plasma medium, as;

3 $${V}_{B}={\left(\frac{{n}_{e}\sqrt{eK{T}_{e}}{H}_{p}^{2}}{{\varepsilon }_{0}}\right)}^{2/3} ,$$

where, ${T}_{e}$ is the electron temperature, and *K* is the Boltzmann constant. The plasma is confined in a glass container. The key tunable parameter is the electron density ${n}_{e}$, which depends on the applied excitation current/power. This yields plasma frequency ${\omega }_{p}$, allowing the plasma elements to behave as highly reflective (when ${\omega }_{p}\gg \omega$, ${\varepsilon }_{r}<0$) to nearly transparent (when ${\omega }_{p}\approx 0$). The Drude model captures strong frequency dependence near the plasma cutoff, enabling wide tunability of the effective surface impedance across the operating band (X/Ku-band in this work).

**Fig. 1:**
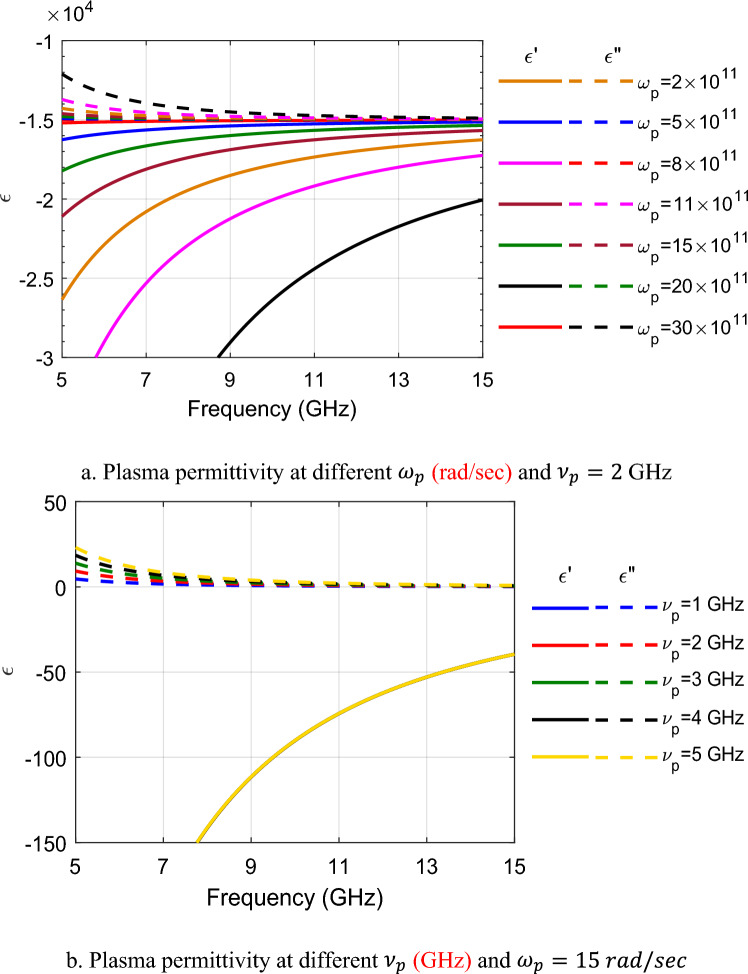
The frequency response of the dispersive permittivity of the plasma material.

Figure 1 illustrates the frequency dependence of the complex relative permittivity (*ε* = *ε'* + *i ε''*) of a plasma modeled using the Drude dispersion model in the microwave range (5–15 GHz). Figure 1a shows the effect of varying the plasma angular frequency ${\omega }_{p}$ (from 2 × 10^11^ to 30 × 10^11^ rad/sec) while keeping the collision frequency fixed at ${\nu }_{p}=2$ GHz. As the frequency increases, the real part ε' rises from large negative values toward zero, indicating a transition from highly reflective (metallic-like) behavior to reduced reflectivity. The imaginary part ε'' decreases toward zero, corresponding to lower absorption losses. Higher ${\omega }_{p}$ values result in more negative ε' and higher ε'' across the band, reflecting denser plasmas with stronger dispersion and greater conductivity/losses. Figure 1b examines the effect of varying the collision frequency ${\nu }_{p}$ (from 1 to 5 GHz) while keeping the plasma frequency fixed ${\omega }_{p}=15 rad/sec$. Increasing the collision frequency has a relatively minor impact on the real part ε' (which remains largely unchanged and strongly negative), but causes a noticeable increase in the imaginary part ε'', indicating higher absorption losses due to enhanced electron scattering.

## Far-field focused hologram reflectarray antenna

**Fig. 2:**
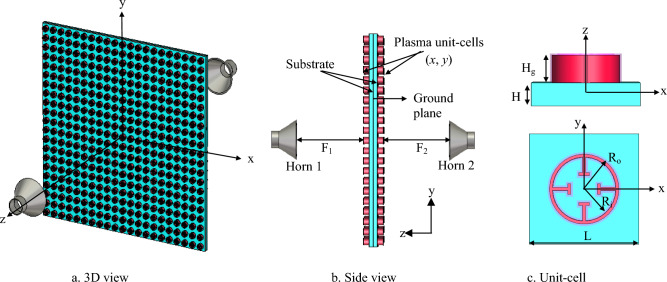
The geometry of the 21 × 21plasma GHRA with unit-cell dimensions L = 15 mm, H = 4 mm, H_g_ = 5 mm​, R_o_ = 5 mm,​ and R_i_ = 4 mm.

**Fig. 3:**
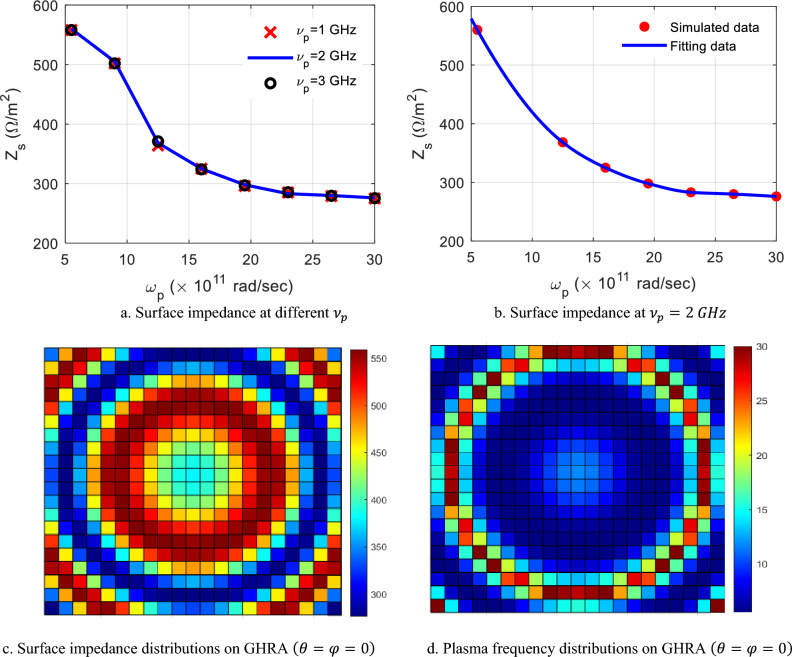
The surface impedance response versus plasma frequency and their distributions over the GHRA at φ = 0° and ϴ = 0°.

Figure 2 depicts a 441-element plasma GHRA in a 21 × 21 rectangular arrangement, with two back-to-back genus hologram surfaces separated by a conducting ground plane, spanning an area of 31.5 × 31.5 cm^2^. Each unit cell consists of a cylindrical ring dielectric tube (*ε*_*r*_ = 3.4, *t*_*g*_ = 0.2 mm) filled with argon gas, characterized by plasma frequency (${\omega }_{p}$) and collision frequency (${\nu }_{p}$). The ring dimensions are optimized for 10 GHz, with four 1.5-mm-wide arms, inner radius *R*_*i*_ = 4 mm, outer radius *R*_*o*_ = 5 mm, and height *H*_*g*_ = 5 mm. Two LP horn antennas (*R*_*H*_ = 27.5 mm, *L*_*H*_ = 46.6 mm, *t*_*H*_ = 2.3 mm) illuminate the GHRA from 31.5 cm, positioned normal to each surface for efficient energy coupling. The reconfigurable plasma’s electrical properties allow precise control of reflection phase and amplitude. Each GHRA surface can be independently activated for EMW radiation, offering versatile beam manipulation from either surface without mutual interference. Each element is independently excited by a low-frequency/DC source connected to electrodes at the tube ends. By varying the discharge voltage, the electron density ${n}_{e}$ is modulated, directly controlling the plasma conductivity and thus the local surface impedance of the holographic metasurface. Bias lines are routed beneath the ground plane to minimize parasitic effects^27^. For plasma uniformity, we assume quasi-uniform electron density along the length of each cylindrical element, which is a valid approximation for DC or low-frequency AC discharges in thin tubes. Radial non-uniformity is neglected, as the tube diameter is much smaller than the wavelength, and full-wave simulations incorporate an effective homogeneous permittivity. These parameters were validated against experimental data from similar plasma tube characterizations reported in the literature. To simulate the GHRA unit cell, the eigenmode solver in CST-MWS is employed to calculate the dispersion characteristics. Subsequently, the frequency-domain solver (or driven modal solver) with periodic boundary conditions is used to determine the variation in surface impedance across different plasma ionization states. Figure 3a plots the simulated surface impedance against plasma frequency $\omega$_*p*_ = 5 × 10^12^ – 30 × 10^12^ rad/sec (with 21 discrete points evaluated), for a fixed frequency of 10 GHz at ${\nu }_{p}$= 1 GHz, 2 GHz, and 3 GHz. A negligible effect on the unit-cell impedance is observed with variations in ${\nu }_{p}$, resulting in only a minor change of approximately 5 Ω/m^2^ across $\omega$_*p*_ variation. The collision frequency is set to 2 GHz (a typical value for low-pressure argon/mercury discharges at a few Torr), which introduces moderate losses but ensures realistic reconfiguration speeds. To establish a mathematical relationship between the surface impedance and the plasma frequency across the entire range for the proposed unit cell, a curve-fitting approach is employed. The impedance response versus $\omega$_*p*_ is modelled using a 3^rd^-order fitting equation, achieving a mean square error (MSE) of 0.98% given by:

4 $${z}_{s}={a}_{1} {\omega }_{p}^{3}+{a}_{2} {\omega }_{p}^{2}-{a}_{3} {\omega }_{p}-826.2$$

where ${a}_{1}$ $=-0.02791, {a}_{2}$ =2.22, and ${a}_{3}$ =59.83 are the coefficient of the fitting equation. The plasma GHRA surface impedance increases monotonically from approximately 267 to 579 Ω for plasma frequency decreases from 30 × 10^12^ rad/sec to 5 × 10^12^ rad/sec (corresponding to *n*_*e*_ = 7.8 × 10^17^–2.8 × 102^1^ m^-3^) due to the reduction in plasma permittivity as shown in Fig. 3b.

The radiation characteristics of the proposed GHRA are determined through a two-step process. First, the surface impedance distribution across the antenna is computed based on the objective beam characteristics, where the impedance is modulated by varying the plasma ionization frequency (i.e., the effective electron density in the plasma tube). Second, a full-wave electromagnetic simulation of the complete antenna structure is performed to accurately predict its far-field radiation patterns, gain, and other performance metrics. First, the hologram is implemented as a modulated surface impedance ${Z}_{s}\left(\mathbf{r}\right)$ on a metasurface. A guided surface wave (reference) interacts with this modulation to produce leaky-wave radiation matching the desired beam (object wave). The surface impedance is typically modulated as a reactance given by^28^:

5 $${Z}_{s}(\mathbf{r})=j[{X}_{o}+M\mathfrak{R}\{{\psi }_{\mathrm{ref}}(\mathbf{r}{)}^{*}\cdot {\psi }_{obj}(\mathbf{r})\}]$$

Simplified for scalar isotropic cases to:

6 $${Z}_{s}(\mathbf{r})=j(\overline{X }+M\mathrm{cos}({\psi }_{obj}(\mathbf{r})-{\psi }_{ref}(\mathbf{r})))$$

where ${X}_{0}$ is the average surface impedance, and *M* is the modulation coefficient. The phase of the reference surface wave from a feed point, ${\psi }_{ref}$ is defined by:

7 $${\psi }_{\mathrm{ref}}= {e}^{-j{k}_{s}r}$$

and the phase of the desired radiated object wave (plane wave for pencil beam) is given by:

8 $${{\psi }_{\mathrm{obj}}=e}^{j{k}_{o}\left(x\mathrm{sin}\theta \mathrm{cos}\phi +y\mathrm{sin}\theta \mathrm{sin}\phi \right)}$$

where *k*_o_ is the free space wave number, *k*_s_ is the surface wave number, (θ, ϕ) are the objective beam direction, and *r* is the radial distance from the feeder to element (*x, y*) on the hologram surface. Using Eq. (6) to Eq. (8), the surface impedance distribution on the GHRA elements is calculated from^28^:

9 $${Z}_{s}(x,y)=j{X}_{o}\left(1+M cos\left[{k}_{s}\sqrt{{x}^{2}+{y}^{2}}-{k}_{o}\left(x \mathrm{sin}\theta \mathrm{cos}\varphi +y \mathrm{sin}\theta \mathrm{sin}\varphi \right)\right]\right)$$

**Fig. 4:**
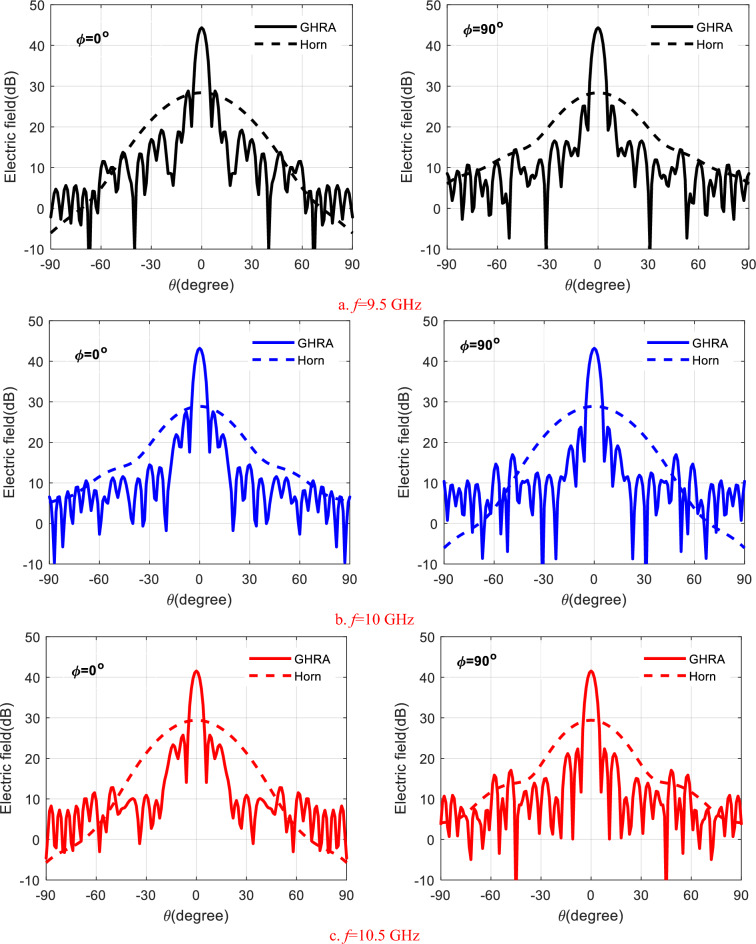
The E-plane and H-plane radiation patterns of a single activated surface of GHRA at different frequencies, 9.5 GHz, 10 GHz, and 10.5 GHz.

**Fig. 5:**
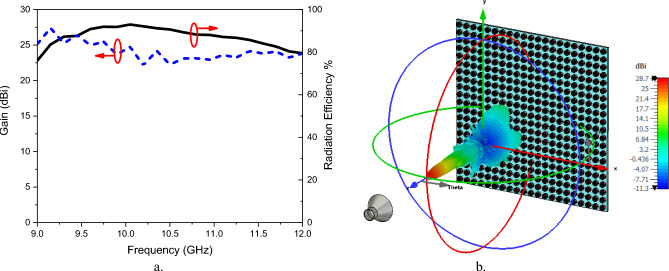
(**a**) Gain variation versus Frequency for GHRA at ($\theta ={0}^{o},\phi ={0}^{o})$, (**b**) The 3D gain pattern at 10 GHz.

Figure 3c illustrates the surface impedance distribution of the plasma GHRA and the corresponding plasma frequency at 10 GHz, with a collision frequency, ${\nu }_{p}=2 GHz$, ($\theta ={0}^{o},\phi ={0}^{o})$*,* ${X}_{0}$=418 Ω, and M = 0.3. The GHRA exhibits quadrant symmetry in the x–y plane, simplifying impedance modeling due to its symmetric structure. Table 1 details the surface impedance values and associated plasma frequencies required to steer the beam at ($\theta ={0}^{o},and \phi ={0}^{o})$ within the first quadrant of the GHRA, enabling precise beam direction control. The CST-MWS full-wave simulator is employed to simulate the radiation characteristics of the GHRA full-structure and two-feeding horns, where open-add space boundaries are employed. The E-plane and H-plane radiation patterns of a single activated surface of GHRA at different frequencies, 9.5 GHz, 10 GHz, and 10.5 GHz, compared with horn antenna only, are shown in Fig. 4. A symmetrical beam is radiated in both planes due to the symmetry of GHRA and the feed position. A slight decrease in the main beam peak with increasing frequency is noticed. At 10 GHz, the peak gain is 27.8 dBi and reduced by 1 dB around the designed frequency, the side lobe level (SLL) is -19.5 dB, and a half-power beamwidth (HPBW) of 4.9° is achieved. The horn antenna introduces a peak gain of 13.5–15 dBi in the frequency band 9.5–10.5 GHz. Figure 5a shows the frequency response of the GHRA peak gain at ($\theta ={0}^{o},\phi ={0}^{o})$. Stable gain is noticed with a peak value of 28.7 dBi and a 1-dB variation bandwidth of 1.1 GHz. The GHRA introduces a radiation efficiency of 83% at 10 GHz, affected by losses in the plasma medium. The 3D gain pattern at 10 GHz is plotted in the Fig. 5b, where a directive pencil beam is noticed with a high front-to-back ratio (FBR) of 30.1 dB.

**Table 1:** The surface impedance and the corresponding plasma frequencies ($\omega$_*p*_ × 10^11^ rad/sec) needed to direct the beam to $\left(\theta =\varphi =0\right)$ of the 1st-quadrant of the GHRA.

Z_s_ = 276 Ω $\omega$_*p*_ × *10*^*11*^ = 30 rad/sec	342.3 Ω 14.4	464.2 Ω 7.7	546.5 Ω 5.6	555.2 Ω 5.5	510.6 Ω 6.2	448.4 Ω 8.4	393.8 Ω 11.2	356.3 Ω 13.3	335.8 Ω 15	329.5 Ω 15.6
342.3 Ω 14.4	478.1 Ω 7.2	556.4 Ω 5.5	536.2 Ω 5.7	456 Ω 8.0	370.9 Ω 12.4	312.1 Ω 17.5	283.9 Ω 23	276.2 Ω 30	277 Ω 29.2	278.2 Ω 28.4
464.2 Ω 7.7	556.4 Ω 5.5	527.0 Ω 5.8	425.1 Ω 9.5	329.5 Ω 15.6	281.5 Ω 25.2	279.8 Ω 27.5	303.6 Ω 18.7	332.2 Ω 15.3	353.0 Ω 13.6	360.4 Ω 13.0
546.5 Ω 5.6	536.2 Ω 5.7	425.1 Ω 9.5	317.6 Ω 16.9	276.2 Ω 30.0	298.9 Ω 19.5	353.0 Ω 13.6	408.2 Ω 10.4	449.4 Ω 8.4	473.2 Ω 7.4	480.8 Ω 7.1
555.2 Ω 5.5	456.0 Ω 8.0	329.5 Ω 15.6	276.2 Ω 30.0	308.6 Ω 18.0	383.7 Ω 11.7	457.5 Ω 8.0	508.8 Ω 6.2	536.7 Ω 5.7	548.7 Ω 5.5	551.7 Ω 5.5
510.6 Ω 6.2	370.9 Ω 12.4	281.5 Ω 25.2	298.9 Ω 19.5	383.7 Ω 11.7	473.2 Ω 7.4	531.9 Ω 5.7	556.5 Ω 5.5	559.6 Ω 5.5	555.3 Ω 5.5	552.9 Ω 5.5
448.4 Ω 8.4	312.1 Ω 17.5	279.8 Ω 27.5	353.0 Ω 13.6	457.5 Ω 8.0	531.9 Ω 5.7	559.2 Ω 5.5	552.9 Ω 5.5	533.6 Ω 5.7	516.7 Ω 6.0	510.3 Ω 6.2
393.8 Ω 11.2	283.9 Ω 23.0	303.6 Ω 18.7	408.2 Ω 10.4	508.8 Ω 6.2	556.5 Ω 5.5	552.9 Ω 5.5	522.8 Ω 5.9	489.2 Ω 6.8	465.5 Ω 7.7	457.2 Ω 8.0
356.3 Ω 13.3	276.2 Ω 30.0	332.2 Ω 15.3	449.4 Ω 8.4	536.7 Ω 5.7	559.6 Ω 5.5	533.6 Ω 5.7	489.2 Ω 6.8	448.8 Ω 8.4	422.8 Ω 9.6	414.0 Ω 10.1
335.8 Ω 15.0	277.0 Ω 29.2	353.0 Ω 13.6	473.2 Ω 7.4	548.7 Ω 5.5	555.3 Ω 5.5	516.7 Ω 6.0	465.5 Ω 7.7	422.8 Ω 9.6	396.5 Ω 11.0	387.9 Ω 11.5
329.5 Ω 15.6	278.2 Ω 28.4	360.4 Ω 13.0	480.8 Ω 7.1	551.7 Ω 5.5	552.9 Ω 5.5	510.3 Ω 6.2	457.2 Ω 8.0	414.0 Ω 10.1	387.9 Ω 11.5	379.4 Ω 11.9

**Fig. 6:**
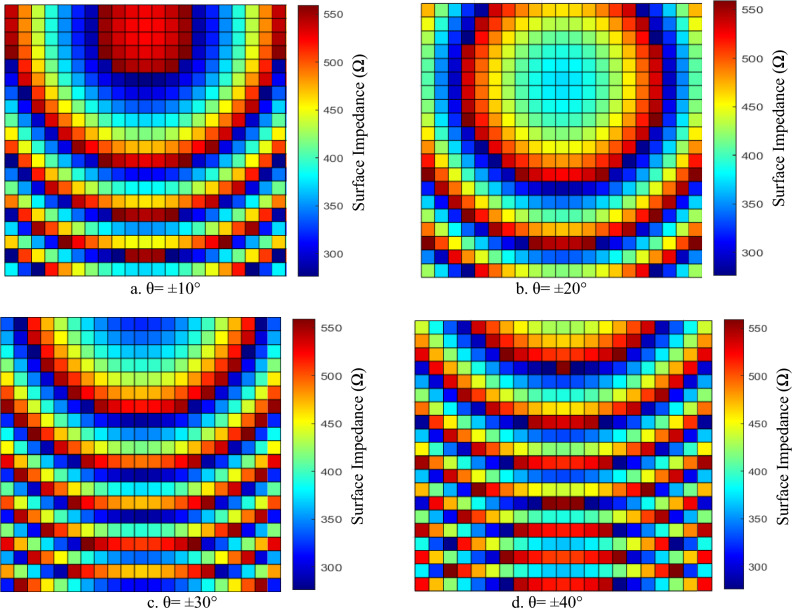
The surface impedance distribution of the single activated surface of GHRA for different beam directions.

The reconfigurability of the proposed GHRA stems from the unique tuning properties of plasma behavior. By applying an external bias voltage across the elements, the electron density in the plasma tubes can be electronically controlled, which alters the surface impedance on the GHRA without any mechanical movement or modification to the physical geometry of the structure. This enables dynamic redistribution of the holographic interference pattern encoded as a spatially varying surface impedance across the entire antenna surface. Consequently, the characteristics of the radiated beam (e.g., pencil beam, scanned beam, or multi-beam patterns) can be electronically reconfigured in real-time simply by adjusting the bias voltage profile, offering fast, low-loss, and highly flexible beam steering and pattern synthesis capabilities. Figure 6 illustrates the calculated surface impedance distributions across the aperture of the GHRA for achieving beam steering in the E-plane ($\varphi =0)$ at different elevation angles (θ). Each subfigure (a–d) depicts the spatially varying surface impedance distribution required to form a directive pencil beam pointed at θ =  ± 10°, ± 20°, ± 30°, and ± 40°, respectively. The impedance patterns exhibit characteristic concentric elliptical/circular fringe-like modulations, which represent the holographic interference pattern between the reference surface wave (launched from the central feed) and the target radiation direction. This tunability leverages the plasma’s reconfigurable electrical properties to control EMW reflection.

**Fig. 7:**
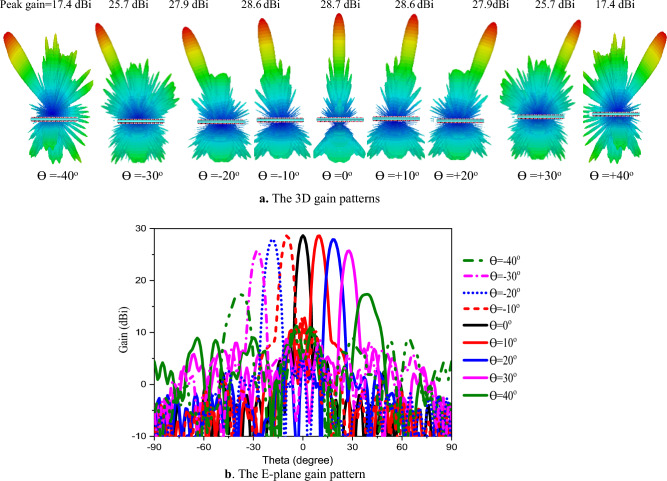
The beam steering patterns for a single activated surface of GHRA at different directions at 10 GHz.

Figure 7a presents the gain patterns for a single activated surface of the GHRA, demonstrating its electronic beam scanning capability at 10 GHz. The GHRA achieves precise beam steering across angles θ =  ± 10°, ± 20°, ± 30°, and ± 40° in the E-plane in Fig. 7b, showcasing a significant advancement in reconfigurable antenna technology. The GHRA maintains symmetry around the x-axis, but y-axis symmetry is intentionally disrupted due to the offset beam radiation strategy. This controlled asymmetry enhances the antenna’s ability to direct beams off-broadside, a critical feature for applications requiring wide-angle scanning, such as satellite communications and radar systems. The maximum gain reaches 28.7 dBi at θ = 0 (broadside), highlighting the GHRA’s high directivity. As the beam deflects to θ =  ± 40°, the gain reduces to 17.4 dBi, a loss of 11.3 dBi, which is expected due to the cosine taper effect in scanned arrays. This performance underscores the GHRA’s ability to maintain substantial gain across a wide scanning range, a notable improvement over conventional reflectarrays that often suffer greater gain degradation at large angles. Back-radiation, resulting from the GHRA’s finite size, is observed, with the highest front-to-back ratio (FBR) of 11.1 dB achieved at θ =  ± 40°. The radiation patterns in the H-plane (ϕ = 90°) are not presented for the beam-steering cases, as the holographic impedance modulation is designed to direct nearly all radiated energy toward the E-plane (ϕ = 0°). This results in highly suppressed radiation in the H-plane, yielding very low cross-polarized fields and negligible sidelobes in the ϕ = 90° plane across the scanned beam directions. This indicates effective energy focusing despite the compact 31.5 × 31.5 cm^2^ footprint, a trade-off that balances size constraints with performance. The ability to maintain a reasonable FBR at extreme scanning angles highlights the GHRA’s practical utility in space-constrained applications. The SLL increases (-19.5 dB to-5 dB) with larger beam scanning angles, accompanied by an increase in HPBW (4.9° to 10°). This behavior is consistent with phased array theory but is mitigated in the GHRA through precise plasma frequency control, which optimizes the surface impedance distribution.

**Fig. 8:**
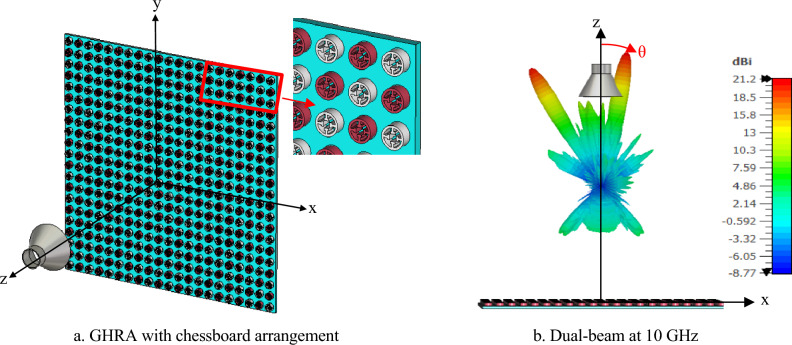
The GHRA structure has a chessboard arrangement with a single surface activated for dual beams at (θ_1_ = 10°, θ_2_ = -30°, φ_1_ = 0°).

**Fig. 9:**
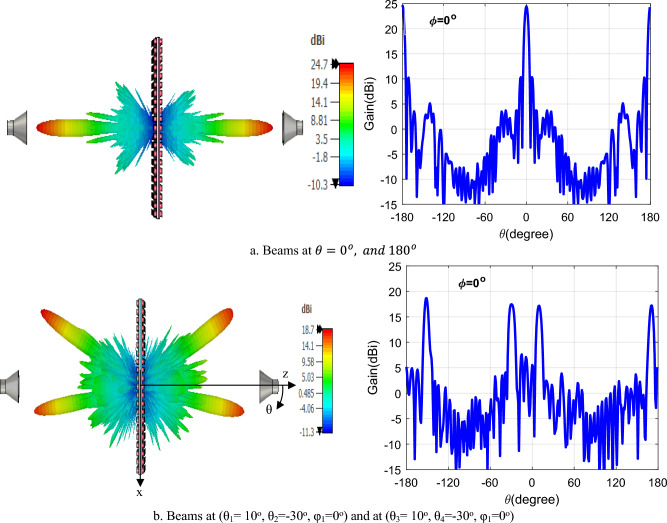
The structure of GHRA has a chessboard arrangement with two surfaces activated for dual beams at 10 GHz.

Figure 8a illustrates the plasma GHRA configured in a chessboard arrangement, enabling dual-beam radiation at 10 GHz. This approach divides the unit cells into two distinct groups, each independently responsible for generating a separate beam. The chessboard pattern represents a groundbreaking method for achieving multi-beam functionality within a single antenna aperture, significantly enhancing the GHRA’s versatility for applications like multi-target tracking and satellite communications. Figure 8b shows the 3D gain patterns for the dual beams radiated at 10 GHz, highlighting the GHRA’s ability to produce precisely directed beams. Beam 1 is directed at (θ₁ = 10°, φ₁ = 0°) with a peak gain of 21.2 dBi, while Beam 2 is directed at (θ₂ = -30°, φ₂ = 0°) with a peak gain of 20.6 dBi. The high gain and directional accuracy of these beams demonstrate the GHRA’s advanced plasma-based impedance modulation, which allows tailored beam steering without mechanical reconfiguration. By dynamically adjusting the plasma frequency of individual unit cells, the GHRA enables customizable beam directions and characteristics. This flexibility allows the antenna to adapt to diverse operational requirements, such as simultaneous communication with multiple receivers, with minimal hardware complexity. Figure 9a depicts the radiation pattern when both GHRA surfaces are ionized, producing directive beams at θ = 0° and θ = 180° with a peak gain of 24.7 dBi and a half-power beamwidth (HPBW) of 6°. This bidirectional radiation capability, achieved through independent ionization of each surface, represents a pioneering approach to maximizing antenna utility. Figure 9b illustrates the GHRA’s ability to radiate dual beams from each surface at 10 GHz. Each surface generates two beams: Beam 1 at (θ₁ = 10°, θ₂ = -30°, φ₁ = 0°) and Beam 2 at (θ₃ = 10°, θ₄ = -30°, φ₁ = 0°), both with a peak gain of 18.7 dBi. The ability to independently control dual beams from each surface, enabled by precise plasma frequency tuning, is a novel feature that enhances the GHRA’s applicability in complex scenarios, such as multi-user communication systems or phased-array radar.

## Design of near-field focused genus hologram reflectarray

Near-field (NF) focusing is an emerging technique in applications such as medical therapy, sensing, inspection, RFID, and microscopy, where precise energy concentration is required. Unlike far-field radiation, NF focusing transforms an incident plane wave into spherical waves that converge at a specific focal point located at a distance, *R*_*o*_*,* from the array aperture. The plasma-based GHRA achieves high-resolution energy focusing, enabling enhanced performance in targeted applications. The transformation from plane to spherical waves in the GHRA is governed by the controlled manipulation of the electromagnetic wave’s phase front. Each unit cell in the GHRA is dynamically tuned via plasma frequency and collision frequency to modulate the surface impedance of each element, effectively shaping the reflected wavefront to converge at the desired focal point. To achieve NF focusing, an additional phase shift is introduced to each element in the GHRA array. This phase shift compensates for the path length differences between the array elements and the focal point, ensuring that the reflected spherical waves align in phase at R_o_ for each element, as calculated from^29^:

10 $${\varphi }_{NF}=\frac{2\pi }{\lambda }\left(\sqrt{{x}^{2}+{y}^{2}+{R}_{o}^{2}}-{R}_{o}\right) \mathrm{radians}$$

This phase shift ensures that the wavefronts from all elements constructively interfere at the focal point, R_o,_ creating a high-intensity focus. The surface impedance modulation of the GHRA given in Eq. (9) is updated to:

11 $${Z}_{s}(x,y)=j{X}_{o}(1+M \mathrm{cos}\left[{k}_{s}\sqrt{{x}^{2}+{y}^{2}}-{k}_{o}\left(x \mathrm{sin}\theta \mathrm{cos}\varphi +y \mathrm{sin}\theta \mathrm{sin}\varphi \right)+{\varphi }_{NF}\right])$$

The NF-focused power distribution at the focal plane R_o_ has 3-dB focused width, W, calculated from^29^:

12 $$W=0.886 {R}_{o}\cdot \frac{{\lambda }_{o}}{L}$$

where *L* = 31.5 cm is the array length, and R_o_ = 63.6 cm. By calculating the required phase shift using Eq. (10) and dynamically adjusting the surface impedance distribution using Eq. (11), the GHRA precisely enables the transformation of incident plane waves into spherical waves, converging at a well-defined focal point along the z-axis.

The Poynting vector is defined as the cross product of the electric field **E** and the complex conjugate of the magnetic field **H** (i.e., **S** = **E** × H*). The real part of the Poynting vector represents the time-averaged active power density (the power that is actually transferred or radiated), while its imaginary part corresponds to the reactive power density (associated with stored energy in the near field)^24^. By introducing appropriate phase shifts across the elements of the GHRA , both the active and reactive power densities can be concentrated at the target focal plane. In this configuration, the ratio of the active power density to the reactive power density is very high, indicating strong dominance of the propagating (active) power over the stored (reactive) component.

Consequently, for this analysis, only the active power density is considered significant and is used in the subsequent calculations and results, and is calculated from^24^:

13 $$S=\Vert Re\left(\overrightarrow{S}\right)\Vert =\Vert Re\left(\overrightarrow{E}\times {\overrightarrow{H}}^{*}\right)\Vert$$

**Fig. 10:**
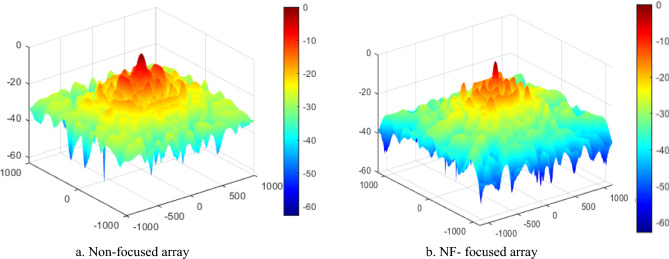
The 3-D normalized power density of GHRA with single surface activated.

**Fig. 11:**
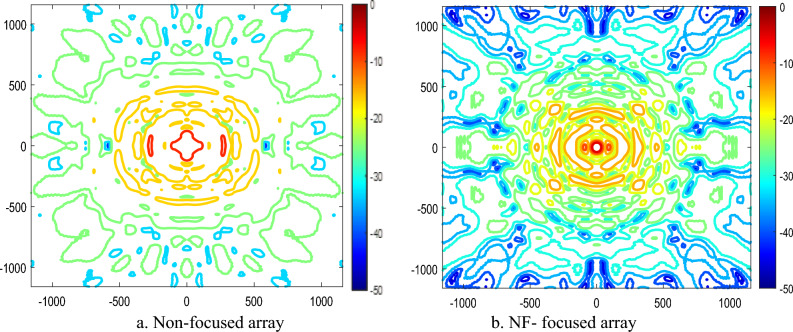
The x–z contour plot of the normalized power density of the GHRA with a single surface activated.

**Fig. 12:**
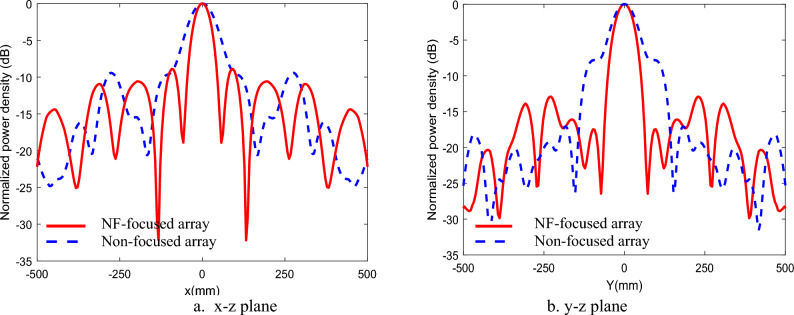
The normalized power density of 21 × 21 GHRA with a single surface activated in different planes.

The equivalent plane wave power densities are defined from the E-field and H-field as follows

14 $${S}_{e}=\frac{{\Vert {\overrightarrow{E}}_{x}\Vert }^{2}+{\Vert {\overrightarrow{E}}_{y}\Vert }^{2}+{\Vert {\overrightarrow{E}}_{z}\Vert }^{2}}{{\eta }_{o}}$$

15 $${S}_{h}={\eta }_{o}\cdot \left({\Vert {\overrightarrow{H}}_{x}\Vert }^{2}+{\Vert {\overrightarrow{H}}_{y}\Vert }^{2}+{\Vert {\overrightarrow{H}}_{z}\Vert }^{2}\right)$$

where *S*_*e*_ and* S*_*h*_ represent the equivalent plane-wave power densities calculated from the electric field (E-field) and magnetic field (H-field), respectively, using the free-space intrinsic impedance η₀ = 377 Ω. In^30^, a comparison is provided between the true active power density in the near field (as given by Eq. (13)) and the power density estimated using the equivalent plane-wave approximations derived from either the E-field or H-field (Eqs. (14) and (15)). It has been shown that, within the near-field (Fresnel) region, the power density computed from the E-field alone is generally sufficient for accurate evaluation of the radiated power density^31^. The near-field performance of the NF-focused GHRA is presented and compared with the corresponding near-field behavior of the non-focused GHRA. The GHRA’s NF focusing exhibits quadrant symmetry across the array aperture, ensuring uniform energy distribution. Figure 10 shows the 3D normalized power density, comparing non-focused and NF-focused patterns for a single activated surface. The focused case achieves a tightly concentrated power spot at R_o_ = 63.6 cm, with a focal spot width of 3.25 cm (major axis) and 3.75 cm (minor axis), as depicted in the x–z plane contour plots of Fig. 11. A notable significant reduction in SLL for the NF-focused array, achieving -17.4 dB in the x–z plane compared to -8.6 dB in the y–z plane, as shown in Fig. 12. This low SLL enhances the antenna’s efficiency by minimizing unwanted radiation, a key milestone in improving the signal-to-noise ratio for NF applications.

**Fig. 13:**
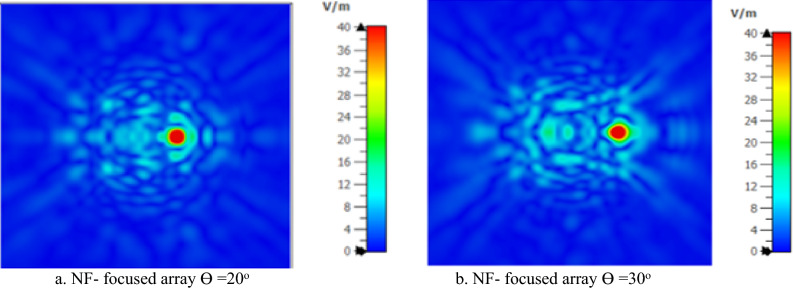
The x–z contour plot of the normalized power density of the GHRA with a single surface activated with an offset focused beam.

**Fig. 14:**
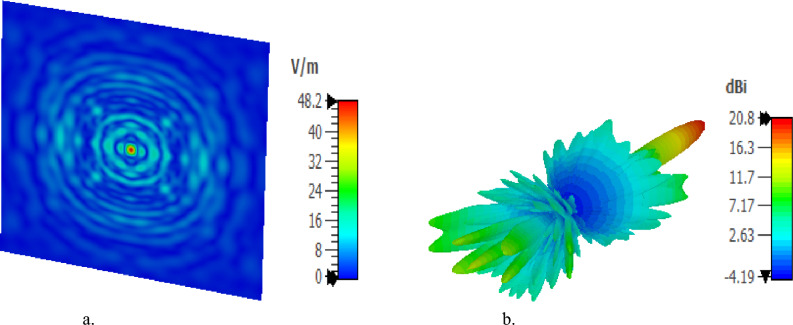
GHRA with two surfaces activated the x–z contour plot of the normalized power density of the plasma GHRA with two surfaces activated.

The GHRA achieves dynamic NF beam steering by adjusting phase distributions, directing the focused beam off-broadside at = 20° and Ɵ = 30°, as illustrated in Fig. 13. Despite the increase in focal spot width with larger deflection angles, the ability to steer the NF focus while maintaining high resolution is a groundbreaking achievement. This flexibility supports applications requiring adaptive focusing, such as inspection systems, where the GHRA’s reconfigurable plasma elements enable real-time beam adjustments without mechanical intervention. A pioneering achievement is the GHRA’s ability to simultaneously support NF focusing and far-field radiation at 10 GHz by activating both surfaces, one for NF focusing and the other for far-field radiation. Figure 14a presents the contour plot of normalized power density in the x–z plane, confirming effective NF focusing, while Fig. 14b displays the 3D far-field radiation pattern with a peak gain of 20.8 dBi. This dual-mode capability, enabled by independent control of each surface’s plasma properties, allows the GHRA to address diverse operational needs within a single compact structure.

Table 2 compares reconfigurable antenna arrays with the proposed genus holographic reflectarray. Large reflectarrays (e.g., 10,240 elements in Ref.^33^) deliver high gain (~ 37 dBi) and wide scan angles (± 60°), suiting tracking/detection systems, while medium-sized transmitarrays^39^ offer moderate gain (~ 22–23 dBi) for satellite and microwave links. Reflectarrays enable large-angle scanning (± 60°) via digital phase control, and transmitarrays support 2D steering (± 50°) for advanced beamforming. Emerging hybrid designs, like the proposed reflectarray, facilitate simultaneous forward/backward beams for enhanced flexibility. Literature arrays employ PIN diodes, phase shifters, and metasurfaces for dynamic control, with metasurface variants enabling polarization/multibeam switching using fewer devices. However, PIN/varactor-based systems suffer RF losses, nonlinearity, distortion, biasing complexity, and radar detectability from metal structures. In contrast, the proposed plasma-based reflectarray boasts low RCS (nearly invisible when off), tunable frequency/pattern/polarization via plasma density, reduced interference/coupling without permanent metals, and rapid switching.

**Table 2:** Comparison of the proposed genus holographic reflectarray antenna with previously reported works.

Ref	No. of Elements	Element Type	No. of Beams/States	Scanning Angle Range	Frequency	Gain	Reconfigurability	Applications
^32^	≥ 256 (large)	Passive unit-cells with PIN phase shifters	–	Digital beamforming	2.3 GHz	19–21.7 dBi	rotating mechanical phase control	5G / mm Wave beamforming
^33^	160 × 64 (10,240)	Reflective elements + PIN diodes	Fast monopulse beams	± 60° (azimuth)	X-band	~ 37.4 dBi	PIN diode phase switching	Tracking & detection
^34^	Large planar RA	Metal groove reflect elements	Multiple steerable beams	± 20°	mmWave	~ 31.7 dBi	Phase digitization	Satellite & high-gain systems
^35^	Not specified (moderate)	Transmitarray with phase-tunable units	2D continuous steering	± 50°	11.8–12.6 GHz	~ 22.76 dBi	Active phase tuning	Satcom/backhaul
^36^	14 × 14	Reconfigurable TRA unit	Dual beams & scan states	± 60°	~ 9.2 GHz	~ 17.4 dBi (Tx), ~ 16.7 dBi (Rx)	PIN diode phase control	Radar & communication links
^37^	Not specified	Polarization-rotating metasurface	Bidirectional + multibeam	± 40°/ ± 22°	X-band / microwave	~ 19–23.6 dBi	Polarization switching/metasurface	Multi-directional links
^38^	Not specified	Dual polarized element + PIN	Bidirectional beams	Full-space scanning	Microwave	–	Transmission & reflection phase control	Full-space beam control
This work	21 × 21 = 441	27.8–17.4	1, 2, 4	(− 40° to + 40°)/NF- focused beam	Argon filled tube	80^o^	Plasma Material	Medical therapy, satellite communications, and phased-array

## Conclusion

This paper investigates the GHRA characteristics based on plasma ionization for dual-mode operation, enabling simultaneous NF focusing and far-field radiation through independent activation of its two surfaces. This dual-mode capability, based on dynamic plasma frequency modulation, enables a multifunctional surface with applications in medical therapy, satellite communications, and radar systems. The GHRA comprises two plasma holographic surfaces, each with 441 elements, arranged back-to-back on a grounded dielectric substrate. Each unit cell is a cylindrical ring-shaped container filled with inert gas, supported by a dielectric substrate. By applying a DC voltage, the gas is ionized into a plasma state, enabling surface impedance variation from 267 Ω to 579 Ω as the plasma frequency shifts from 5 × 10^12^ rad/sec to 30 × 10^12^ rad/sec. Activating a single GHRA surface allows electronic beam steering from ${\theta }_{o}={-40}^{o} to {\theta }_{o}={+40}^{o},$ without altering the physical structure. A peak gain of 28.7 dBi is achieved at ${\theta }_{o}=0$, and 17.4 dBi at ${\theta }_{o}={\pm 40}^{o}$ with an FBR of 11.1 dB, and SLL is 6.1 dB at ${\theta }_{o}={\pm 40}^{o}$ , reflecting the impact of beam deflection from broadside. Dual-beams are radiated beams from each surface of GHRA with beam 1 at (θ_1_ = 10°, θ_2_ = -30^o °^, φ_1_ = 0°) and beam 2 at (θ_3_ = 10°, θ_4_ = -30^o °^, φ_1_ = 0°), both yielding a peak gain of 18.7 dBi. Near-field (NF) focusing is studied at various directions from the array aperture, with an additional phase shift applied to the elements based on the focal distance. For ${\theta }_{o}={0}^{o},$ a focused spot occurs at R_o_ = 63.6 cm with a width of 3.25 cm for the major axis and 3.75 cm for the minor axis for a single surface activated. Offset NF focusing on ${\theta }_{o}={10}^{o} and {20}^{o}$ produces focal spots with widths of 2.2 cm and 2.5 cm, respectively, for one or both surfaces activated, showcasing the GHRA’s precision in energy concentration for applications such as medical therapy and sensing.

## Data Availability

There are no supplementary materials, and the data is available upon reasonable request.

## Code availability

The program is available upon request.

## References

[1] Rahman, M. S. U., Abou-Khousa, M. A. & Akbar, M. F. A review on microwave non-destructive testing (NDT) of composites. *Eng. Sci. Technol. Int. J.*10.1016/j.jestch.2024.101848 (2024).

[2] Oloumi, D., Winter, R. S., Kordzadeh, A., Boulanger, P. & Rambabu, K. Microwave imaging of breast tumor using time-domain UWB circular-SAR technique. *IEEE Trans. Med. Imaging.*10.1109/TMI.2019.2937762 (2020). 31478843 10.1109/TMI.2019.2937762

[3] Benítez, B. B., Tirado-Mendez, J. A., Tirado-Mendez, J. A. & Jardon-Aguilar, H. An overview of UWB antennas for microwave imaging systems for cancer detection purposes. *Prog. Electromagn. Res. B* **80**, 173–198 (2018).

[4] Zainud‑Deen, S. H., Malhat, H. A., El‑Refaey, E. A. & Badawy, M. M. Genus plasma‑based self‑complementary reconfigurable intelligent metasurfaces. *Plasmonic*10.1007/s11468-024-02215-6 (2024).

[5] Saifullah, Y., He, Y., Boag, A., Yang, G. & Xu, F. Recent progress in reconfigurable and intelligent metasurfaces: A comprehensive review of tuning mechanisms, hardware designs, and applications. *Adv. Sci.*10.1002/advs.202203747 (2022). 10.1002/advs.202203747 PMC9685480 36117118

[6] N. R. Vinayagam K, "Design of multiband frequency reconfigurable antenna with switchable polarization states," Microw Opt Technol Lett. , vol. 66, no. 2, 2024.

[7] T. Anderson, "Plasma Antennas," in Selected Topics in Plasma Physics, IntechOpen, 2020.

[8] Malhat, H. A., Badawy, M. M., Zainud-Deen, S. H. & Awadalla, K. H. Dual-mode plasma reflectarray/ transmitarray antennas. *IEEE Trans. Plasma Sci.* **43**(10), 3582–3589 (2015).

[9] Wang, C., Yuan, B., Shi, W. & Mao, J. Low-profile broadband plasma antenna for naval communications in VHF and UHF bands. *IEEE Trans. Antennas Propag.* **68**(6), 4271–4282 (2020).

[10] Malhat, H. A., Gaber, S. M., Awadall, aK. H. & Zainud-Deen, S. H. Beam steering plasma reflectarray/transmitarray antennas. *Plasmonics* **9**, 477–483 (2014).

[11] Malhat, H. A. & Zainud-Deen, S. H. Dual/circular polarization beam shaping of time-modulated plasma-based magneto-electric dipole antenna arrays. *Opt. Quant. Electron.*10.1007/s11082-021-03482-x (2022).

[12] Zainud-Deen, S. H. et al. Genus plasma-based self-complementary reconfigurable intelligent metasurfaces. *Plasmonics* (2024).

[13] Pandi, S., Balanis, C. A. & Birtcher, C. R. Design of scalar impedance holographic metasurfaces for antenna beam formation with desired polarization. *IEEE Trans. Antennas Propag.* **63**(7), 3016–3024 (2015).

[14] Z. Li, W. Cui, R. Liu, M. Wang, C. Fan, H. Zheng, and E. Li, “Metantenna design with one-dimensional holographic concept,” International Journal of RF and Microwave Computer Adid Engineering, vol. 31, no. 3, pp. 1–12, 2021. R. Zhao, L. Huang, and Yongtian Wang, "Recent advances in multi-dimensional metasurfaces holographic technologies," PhotoniX, pp. 1–20, 2020.

[15] Shang, G. et al. Metasurface holography in the microwave regime. *Photonics* **8**(5), 1–18 (2021).

[16] H. Jiaqi, L. Long, T. Shuncheng, M. Xiangjin, F. Qiang, L. Haixia, Z. , Yu, and L. Guisheng, Frequency-diverse holographic metasurface antenna for near-field microwave computational imaging. Front. Mater. vol. 8, pp. 1–8, 2021.

[17] Yurduseven, O., Marks, D. L., Fromenteze, T. & Smith, D. R. Dynamically reconfigurable holographic metasurface apreture for a Mills-cross monochromatic microwave camera. *Opt. Express* **26**, 5281–5291 (2018). 29529733 10.1364/OE.26.005281

[18] Huang, L., Zhang, S. & Zentgraf, T. Metasurface holography: From fundamentals. *Nanophotonics* **6**, 1169–1190 (2018).

[19] Jia, Y., Liu, Y., Feng, Y. & Zhou, Z. Low-RCS holographic antenna with enhanced gain based on frequency-selective absorber. *IEEE Trans. Antennas Propag.* **68**(9), 6516–6526 (2020).

[20] Karimipour, M., Komjani, N. & Aryanian, I. Shaping electromagnetic waves with flexible and continuous control of the beam directions using holography and convolution theorem. *Sci. Rep.* **9**, 1–13 (2019). 31413284 10.1038/s41598-019-48301-2 PMC6694119

[21] Jiang, Q., Cao, L., Huang, L., Heb, Z. & Jin, G. A complex-amplitude hologram using an ultrathin. *Nanoscale* **12**, 24162–24168 (2020). 33245308 10.1039/d0nr06461k

[22] Zhao, W. et al. Full-color hologram using spatial multiplexing of dielectric metasurface. *Opt. Lett.* **41**(1), 147–150 (2016). 26696180 10.1364/OL.41.000147

[23] Smith, D. R. et al. An analysis of beamed wireless power transfer in the Fresnel zone. *J. Appl. Phys.* **121**, 014901 (2017).

[24] S. H. Zainud-Deen, S. M. Gaber, H. A. Malhat, and K. H. Awadalla, “Multilayer Dielectric Resonator Antenna Transmitarray for Near-Field and Far-Field Fixed RFID Reader,” 29^th^ national radio conference (NRSC 2012), Cairo, Egypt, 2012.

[25] Damavandi, M. A. & Khalaj-Amirhosseini, M. Homogeneous microwave near-field power focusing using a cylindrical antenna array. *Sci. Rep.* **13**, 14698 (2023). 37679458 10.1038/s41598-023-41866-z PMC10484950

[26] Dassault Systèmes. CST Microwave Studio Suite 2021.

[27] Magarotto, M. et al. Plasma antennas: A comprehensive review. *IEEE Access* **12**, 80468–80490 (2024).

[28] N. A. Eltersy, H. A. Malhat and S. H. Zainud-Deen, Dual-beam conformal hologram metasurface leaky wave antenna based on surface impedance modulation. 2023 40th National Radio Science Conference (NRSC), Giza, Egypt, 2023, pp. 9–16.

[29] H. Nakano et al., Near-field focusing of aperture antennas. IEEE Trans. Antennas Propag. vol. 34, no. 6, 1986.

[30] Y. Adane, M.F. Wong, C. Dale, A. Gati, J. Wiart, and V.F. Hanna, “Near field power density characterization of radio base station antennas using spherical harmonics optimization techniques,” European Conference on Wireless Technology, Amsterdam, pp. 121–124, 2004.

[31] Fenn, A. J. On the radial component of the electric field for a monopole phased array antenna focused in the near zone. *IEEE Trans. Antennas Propag.* **40**(6), 723–727 (1992).

[32] Ahn, B. et al. Wide-angle scanning phased array antenna using high gain pattern reconfigurable antenna elements. *Sci. Rep.* **9**, 18391 (2019). 31804507 10.1038/s41598-019-54120-2 PMC6895243

[33] Dai, L. et al. Reconfigurable intelligent surface-based wireless communications: Antenna design, prototyping, and experimental results. *IEEE Access* **8**, 45913–45923 (2020).

[34] Budhu, J., Hum, S. V., Ellingson, S. & Buehrer, R. M. Design of rim-located reconfigurable reflectarrays for interference mitigation in reflector antennas. *IEEE Trans. Antennas Propag.* **72**(4), 3736–3741 (2024).

[35] Yi, M., Bae, Y., Yoo, S. & So, J. Digitized reconfigurable metal reflectarray surfaces for millimeter-wave beam-engineering. *Appl. Sci.*10.3390/app11135811 (2021).

[36] Sun, M. et al. Reconfigurable transmitarray based on frequency selective surface for 2D wide-angle beam steering. *Electronics*10.3390/electronics12183854 (2023).

[37] Hum, S. V. & Perruisseau-Carrier, J. Reconfigurable reflectarrays and array lenses for dynamic antenna beam control: A review. *IEEE Trans. Antennas Propag.* **62**(1), 183–198 (2014).

[38] Qin, F. et al. Multibeam hybrid transmitarray based on polarization rotating metasurface with reconfigurable bidirectional radiation. *IEEE Trans. Antennas Propag.* **72**(9), 6992–7004 (2024).

[39] Tian, X. & Song, L. Dual-polarized bidirectional beam-steering array with beam-scanning capability in full-space. *AEU Int. J. Electron. Commun.*10.1016/j.aeue.2024.155225 (2024).

